# Human development associated with environmental quality in China

**DOI:** 10.1371/journal.pone.0246677

**Published:** 2021-02-11

**Authors:** Xiaoyu Li, Lan Xu

**Affiliations:** 1 Research Center for Economy of Upper Reaches of the Yangtze River, Chongqing Technology and Business University, Chongqing, China; 2 School of Accounting, Chongqing Technology and Business University, Chongqing, China; 3 School of Economics and Business Administration, Chongqing University, Chongqing, China; China University of Mining and Technology, CHINA

## Abstract

This paper aims to investigate the connection between overall environmental quality and human development. Based on China’s provincial panel data from 2004 to 2017, this study constructed the Environment Degradation Index (*EDI*) and Human Development Index (*HDI*) to measure environmental pollution and human development, respectively, and it used the Simultaneous Equations Model (SEM) to assess the relationship between them. The results showed that there was an inverted U-shaped relationship found between *EDI* and *HDI*, and the coefficients of the first and second power of *HDI* were 5.2781 and -2.3476, respectively. Meanwhile, the results also confirmed that environmental pollution, in turn, delayed regional economic growth, and every 0.01 unit increase in *EDI* was correlated with a 3.15% decrease in GDP per capita. It is recommended that the government should speed up human development to surpass the turning point of the inverted U-shaped curve soonest possible.

## 1 Introduction

The traditional environmental Kuznets curve (EKC) depicts the relationship between economic development and a specific type of pollutant, which does not reflect how environmental quality overall changes with human development. On the one hand, the development of human society not only requires economic growth, but it also demands progress in other aspects of life like education and medical treatment. On the other hand, environmental quality is affected by many kinds of pollutants, such as air pollution, water pollution, and solid pollution, and a single pollution index cannot reflect environmental status fully [1]. Therefore, to understand the entire set of characteristics of environmental quality at different stages of human development, it is vital to building composite indicators for both environmental pollution and human development to explore how emission pollution changes with human development.

Although China’s economic and social development has made great progress in recent decades, it has also led to the deterioration of environmental quality, which poses a threat to sustainable development. Environmental problems that appeared in developed countries during the industrialization process in the past hundred years are now prevalent in China, which exhibit structural, compound, and compression characteristics. Both Chinese people and the government have realized the harm caused by pollution and have already begun to pay attention to environmental protection. The government has taken a series of measures to control emissions, which included making stronger environmental regulations, adjusting production and consumption structures, and introducing advanced technologies. These changes may eventually reverse the trend of environmental deterioration. Thus, this paper aims to determine whether there is a turning point for environmental pollution in the process of human development. By using the Environment Degradation Index (*EDI*) and the Human Development Index (*HDI*) to measure comprehensively environmental quality and human development, respectively, this study utilized the Simultaneous Equations Model (SEM) to examine the relationship between *EDI* and *HDI* empirically. The results suggested that there was an inverted U-shaped relationship between human development and environmental pollution, and the worsening of environmental pollution, in turn, delayed regional economic growth.

This paper contributes to the literature in two aspects. First, unlike the traditional EKC curve that studies how specific environmental pollution changes with economic development, this paper explored how general environmental quality varied with human development, which can better explain the relationship between human activity and the natural environment. Second, it uses the Simultaneous Equations Model (SEM) to estimate the parameters, which not only dealt with the problem of endogeneity but also provided insights into the relationship among human development, environmental quality, and economic development.

The remainder of the paper proceeds as follows. The second part reviews the existing literature. The third part constructs indicators for human development and environmental pollution and introduces the framework of the research method. The fourth part is the results and sensitivity analysis, and the last part gives conclusions and policy implications.

## 2 Literature review

### 2.1 The relationship between economic growth and environmental quality

Among existing studies on the relationship between economic growth and environmental quality, the most typical model used is the environmental Kuznets curve (EKC). The EKC hypothesis states that at the beginning of economic development, the government and market participants only focus on the growth of output and income, which leads to the overexploitation of natural resources and an increase in environmental pollution. However, when the economy surpasses a certain turning point, people’s demands for environmental quality rise and they would urge the government to take measures to deal with pollution, such as changing the mode of economic growth and adjusting industrial structure. Thus, there exists an inverted U-shaped relationship between economic growth and environmental pollution [2,3]. Recently, scholars have also added other variables to the EKC model, which include international trade [4,5], industrial structure [6,7], education [8], political democracy [9], and social corruption [10,11]. In contrast, other scholars use different pollutants and different quantitative analytical methods to further test the EKC hypothesis [12,13]. However, due to different modes in economic development and industrial structures, there does not always exist a single form of relationship within EKC that applies to all countries and all pollutants.

Meanwhile, the deterioration of the environment and excessive consumption of resources also influence economic growth. On the one hand, the exhaustibility of resources restricts the sustainability of an economy [14–16]. On the other hand, people’s demand for environmental quality increases with economic development, which, in turn, affects economic growth [17]. Besides, the worsening of environmental pollution also has an impact on output and consumption, which often exhibits negative marginal utility and positive marginal output [18,19].

### 2.2 The relationship between human development and environmental pollution

Human development can be seen as a process in which people expand the real freedoms they enjoy [20]. Progress in human development promotes people’s educational level, and people with higher educational levels not only pursue material wealth, but also require a better living environment. Brasington and Hite [21] found that there was a complementarity between education and environmental quality in the United States. They indicated that when the accumulation of material wealth came at the cost of the environment and resources, the improvement in education caused people to exercise internal restraints from excessive resource demand. One study of an Indian city by Jalan et al. [22] showed that when the educational level of women in a family rose from 0 years to 17 years, the willingness to pay for higher water quality increased significantly from 66 rupees to 144 rupees.

Moreover, based on cross-sectional data on 174 countries, Jha and Bhanu [23] found an inverted N-shaped curve between *EDI* and *HDI*. With a similar econometric model and data from 1990 to 2004, Mukherjee [24] proved that there were nonlinear relationships between human development and environmental pollution in India, which varied with different pollutants. Costantini and Monni [25] analyzed cross-sectional data on 179 countries and found that achieving sustainable economic growth by investing in human capital did not harm the environment. Serkan [26] used data from 15 countries in the Mediterranean region for 1970–2006 that showed that human development reduced regional pollution emissions. Sapci and Shogren [27] used county-level panel data for 2007–2009 in the United States to explore the impact of air quality on human capital. Their study found that for every 1% reduction in pollution, human capital stock increased by 0.10%. In conclusion, human development is an effective way to achieve sustainable economic and environmental development under the constraints of physical resources.

In recent years, many scholars have studied China’s economic and environmental issues. Fan et al. [28] and Ji et al. [29] discussed the relationship between environmental resources and China’s economy from the perspective of resource endowment. Chang et al. [18] and Zheng et al. [30] studied the effect of air pollution on people’s happiness and labor productivity in China. In addition, they have also tested China’s environmental Kuznets curve to verify whether there is a turning point for China’s environmental pollution [31–35]. Some studies also discussed China’s environmental issues from the perspective of foreign direct investment [36,37], environmental regulation [38,39], and income distribution [40,41].

In conclusion, although studies were conducted on the relationship between the economy and the environment in China, most of them focused on economic growth. In contrast, our paper emphasizes China’s environmental pollution through human development. It analyses the correlation between human development and environmental pollution based on the construction and measurement of a composite *HDI* and *EDI*. The targeted aim of this study is to provide a more comprehensive understanding of factors that influence environmental quality in China.

## 3 Materials and methods

### 3.1 Model setting

The traditional EKC hypothesis explains how environmental quality changes with economic development by using GDP per capita to measure economic development. However, this paper aims to explore the non-linear relationship between *EDI* and *HDI*, where *HDI* contains three aspects of human society—life expectancy, average years of education, and per capita income, which can better reflect the status of the economy and human activity [23]. Following the modified EKC model of Jha and Murthy [23], we used *HDI* to replace GDP and constructed the regression model as:

$$EDI_{it}=\beta_{1}HDI_{it}+\beta_{2}HDI_{it}^{2}+\beta_{3}Growth{\;}_{it}+\beta_{4}COND_{it}+\alpha_{i}+\varepsilon_{it}$$ (1)

where *i* and *t* indicate the subscripts of province and time, respectively. *EDI*_*it*_ and *HDI*_*it*_ stand for environmental pollution and human development, respectively. *Growth*_*it*_ is per capita income. *COND*_*it*_ symbolizes other control variables that also affect environmental quality, and *α*_*i*_ is the regional fixed effect.

Besides, change in environmental quality, in turn, affects economic growth [15,19]. One reason for this is that with the aggravation of environmental pollution, people realize the harm caused by it and turn to higher environmental regulations, which affect economic growth by influencing enterprises’ environmental costs, technological innovation, and resource utilization [17,42,43]. For another reason, environmental pollution reduces labor productivity and labor supply [18,19], which would also inhibit economic growth. Therefore, *Growth*_*it*_ is an endogenous explanatory variable in the equation above, and the coefficients would be biased if we estimate them directly. Considering the mutual influence between environmental pollution and economic growth, this paper further introduces another equation to build a simultaneous equation model, which is an effective way to solve the problem of endogeneity. Specifically, based on the C-D production function, we took *Growth* as the explained variable and *EDI* as one of the explanatory variables to establish the following econometric model:

$$Growth_{it}=\gamma_{1}EDI_{it}+\gamma_{2}k_{it}+\gamma_{3}H_{it}+\lambda_{i}+\xi_{it}$$ (2)

where *Growth*_*it*_ is measured by GDP per capita, and *k* represents capital stock per capita. *H*_*it*_ stands for human capital and *λ*_*i*_ is the regional fixed effect. Eqs (1) and (2) constitute the simultaneous equation model that we used to evaluate the effect of human development on environmental pollution.

The control variables in Eq (1) include industrial structure (*Strcu*), technological progress (*Tech*), pollution control intensity (*Inst*), and foreign trade (*Fore*). When an economy is in the take-off and acceleration stage, the acceleration of industrialization and the development of secondary industries bring about serious environmental problems. When the driving force of economic growth turns to low pollution and high output sectors (e.g., services and information), the impact of the economy on the environment becomes favorable to environmental quality. Hence, we introduced the industrial structure (*Strcu*) into the equation, which was measured by the proportion of the output value of the second industry [44,45]. In addition, with the development of the economy, people increase investment in environmental protection technology and R&D, which allows pollution control to become easier. Thus, this study used technological progress (*Tech*) and pollution control intensity (*Inst*) as control variables, which were measured by the natural logarithm of R&D expenditure and the proportion of investment to control environmental pollution in GDP. Furthermore, international trade also influences the environment [46]. Therefore, we also took foreign trade (*Fore*) as one of the control variables and used the ratio of foreign capital to GDP to measure it.

Control variables in Eq (2) include capital per capita (*k*) and human capital (*H*). Referring to Zhang et al. [47], we used the perpetual inventory method to estimate the total amount of capital, and we chose 1952 as the base year because the earlier the base year, the smaller impact is the estimation error. Also, we used an average year of education to measure human capital, and the number of years of education for primary school, junior high school, senior high school, and junior college were 6, 9, 12, and 16, respectively. Finally, *Growth* was measured by GDP per capita. *k*, *H*, and *Growth* all appear in their logarithmic forms.

### 3.2 Estimation method

Due to the existence of endogeneity and heteroscedasticity, our study used the three-stage least square method (3SLS) to estimate simultaneous equations. In the first stage, a simplified form of the simultaneous equation system was estimated. In the second stage, the fitted values of endogenous variables were used to get the 2SLS estimates for all equations. According to the seemingly unrelated regression (SUR) technique, a residual value for each equation was used to estimate the variance and covariance between the equations after the parameters for 2SLS were obtained. In the third stage, a parameter estimator of the generalized least square was obtained. 3SLS produced more effective estimators than 2SLS because it considered the correlation between equations. In a balanced system, the estimators obtained by 3SLS are:

$${\hat{\Delta }}_{3SLS}={\left[\hat{X}\prime (\sum^{{}^{-1}}⊗I_{t})\hat{X}\right]}^{-1}\hat{X}\prime ({\hat{\sum }}^{{}^{-1}}⊗I_{t})Y$$ (3)

$$\hat{X}=[\begin{array}{cccc} Z{\left(Z^{\prime }Z\right)}^{-1}Z^{\prime }X_{1} & 0 & 0 & 0\\ 0 & Z{\left(Z^{\prime }Z\right)}^{-1}Z^{\prime }X_{2} & \ddots & 0\\ \vdots & \ddots & \ddots & \vdots \\ 0 & \ldots & 0 & Z{\left(Z^{\prime }Z\right)}^{-1}Z^{\prime }X_{k} \end{array}]$$ (4)

Where, ∑ is the covariance matrix of the residuals; operator⊗represents the Kronecker product; *Z* is the pre-variable matrix; *X*_*i*_ stands for the *T*×*k*_*i*_ order explanatory variable matrix of equation *i*.

The problem of model identification must be considered before model estimation, that is, whether parameters estimation can obtain from the estimated induced coefficients. The structural form of the simultaneous equations of this study is:

BY+ΓZ=u (5)

where the variable matrix and the coefficient matrix are:

$$Y_{t}=[\begin{array}{c} EDI_{t}\\ Growth{\;}_{t} \end{array}],\;t=1,2,3,\cdots ,T$$ (6)

$$Z_{t}={\left(1,HDIM_{t},HDIM_{t}^{2},HDIM_{t}^{3},COND_{t},k_{t},H_{t},L_{t}\right)}^{\prime },\;t=1,2,3,\cdots ,T$$ (7)

$$u_{t}=[\begin{array}{l} u_{1t}\\ u_{2t} \end{array}],\;t=1,2,3,\cdots ,T$$ (8)

$$\left(B,\Gamma )=[\begin{array}{ccccccccc} 1 & -\beta_{4} & -\beta_{0} & -\beta_{1} & -\beta_{2} & -\beta_{3} & -\beta_{5} & 0 & 0\\ -\gamma_{1} & 1 & -\gamma_{0} & 0 & 0 & 0 & 0 & -\gamma_{2} & -\gamma_{3} \end{array}\right]$$ (9)

In the simultaneous equations, *Growth* and *EDI* are used as endogenous variables. The others are predetermined variables that are assigned by external conditions. According to the order condition and rank condition of model identification, we have:

$$rank(B_{0},\Gamma_{0})=k-1=1$$ (10)

where *k* is the number of endogenous variables. For the number of predetermined variables (*g*) of all equations, we have:

$$g-g_{i}>k_{i}-1$$ (11)

It can be seen that the empirical model in this paper is over-identified, which means the coefficients can be estimated. Besides, we also conducted a specification test with the use of the Husman test to further examine the simultaneous problem of the equations. Specifically, Specifically, we took *Growth*_*it*_ as the explained variable and performed a regression on the pre-determined variables to obtain the estimated value $Growt{\hat{h}}_{t}$ and estimated residual ${\hat{U}}_{it}\cdot (Growth{\;}_{it}-Growt{\hat{h}}_{it})$. Then, we took *EDI* as the explained variable to regress on $Growt{\hat{h}}_{it}$ and ${\hat{U}}_{it}$ and performed a T-test on the coefficient of ${\hat{U}}_{it}$. The result showed that the coefficient of ${\hat{U}}_{it}$ was significant at the 1% level, which means that there exists an interactive relationship between *Growth* and *EDI*, and the estimated parameters would be biased and inconsistent if we use the ordinary least squares as the estimation method. Therefore, the three-stage least square method (3SLS) is suitable for estimating the simultaneous equations in this paper.

### 3.3 Data sources and description

Based on the *Human Development Indices and Indicators—2018 Statistical Update* released by UNDP, we used the geometric average of the following three dimensions to measure *HDI*: i) health level, which was measured by life expectancy at birth. The life expectancy data for 1982, 1990, 2000 and 2010 come from the National Bureau of Statistics of China., and the data for other years were estimated by interpolation and extrapolation. ii) educational level, which was measured by years of education, and iii) living standard, which was measured by per capita national income. UNDP used the per capita income to measure the living standard. However, because of the lack of statistics during the sample period, we used the ratio between per capita GDP at the national level and GNP measured by PPP dollar price in 2011 to estimate the per capita GNP of each province. The calculation method for the sub-indicators and *HDI* are presented by the Eqs (12) and (13).

$${\text{HX}}_{\text{ij}}=({\text{X}}_{\text{ij}}-{\text{X}}_{i}^{*})({\text{X}}_{i}^{**}-{\text{X}}_{i}^{*})$$ (12)

$${\text{HDI}}_{\text{j}}={\left(HX_{1j}*HX_{2j}*HX_{3j}\right)}^{1/3}$$ (13)

Among them, *HX*_*ij*_ and *HDI*_*j*_ represent the ith-dimension of province *j* and Human Development Index of province *j*. *X*_*ij*_ is the actual value of dimension *i* province *j*. *X*_i_* and *X*_i_** represent the minimum and maximum value of dimension *i*, respectively. The higher the value of *HDI*, the higher the level of human development.

*HDI* values and the ranking of the provinces’ representative years during the sample period of 2004–2017 showed that China’s human development level improved, and the average *HDI* value increased from 0.6322 in 2004 to 0.7374 in 2017 (Table 1). However, the relative ranking among provinces remained almost unchanged. Cities in the eastern region, such as Beijing, Shanghai, Tianjin, and Jiangsu, enjoyed a relatively higher level of human development, but cities in the western region, such as Tibet, Guizhou, Yunnan, Qinghai, and Gansu, had a relatively lower level of human development.

**Table 1:** Human Development Index (*HDI*) in 2004, 2010, and 2017 and the average *HDI* during 2004–2017.

Region	Province(city)	2004	2010	2017	Average (2004–2017)
		*HDI*(ranking)	*HDI*(ranking)	*HDI*(ranking)	*HDI*(ranking)
Northeast	Liaoning	0.6781(05)	0.7376(05)	0.7587(07)	0.7331(05)
	Jinlin	0.6579(08)	0.7172(10)	0.7497(12)	0.7097(10)
	Heilongjiang	0.6530(10)	0.7051(12)	0.7318(18)	0.7003(12)
East	Beijing	0.7844(01)	0.8324(01)	0.8859(01)	0.8352(01)
	Tianjin	0.7413(03)	0.7930(03)	0.8413(03)	0.7938(03)
	Hebei	0.6489(11)	0.6959(16)	0.7209(21)	0.6879(17)
	Shanghai	0.7831(02)	0.8114(02)	0.8585(02)	0.8157(02)
	Jiangsu	0.6642(07)	0.7409(04)	0.7836(04)	0.7344(04)
	Zhejiang	0.6805(04)	0.7299(07)	0.7731(06)	0.7307(06)
	Fujian	0.6405(12)	0.7148(11)	0.7565(08)	0.7025(11)
	Shandong	0.6577(09)	0.7177(09)	0.7523(09)	0.7117(08)
	Guangdong	0.6733(06)	0.7342(06)	0.7738(05)	0.7295(07)
	Hainan	0.6404(13)	0.6926(17)	0.7436(14)	0.6902(16)
West	Inner Mongolia	0.6372(15)	0.7224(08)	0.7512(10)	0.7114(09)
	Guangxi	0.6041(24)	0.6685(24)	0.7026(26)	0.6622(24)
	Chongqing	0.6103(21)	0.6882(18)	0.7501(11)	0.6850(18)
	Sichuan	0.5956(26)	0.6611(25)	0.7104(23)	0.6553(26)
	Guizhou	0.5302(30)	0.6017(30)	0.6745(28)	0.6032(30)
	Yunnan	0.5506(29)	0.6080(29)	0.6627(30)	0.6062(29)
	Tibet	0.4778(31)	0.5375(31)	0.5797(31)	0.5209(31)
	Shaanxi	0.6189(19)	0.6987(14)	0.7420(15)	0.6903(15)
	Gansu	0.5677(27)	0.6318(28)	0.6751(27)	0.6249(28)
	Gansu	0.5636(28)	0.6329(27)	0.6722(29)	0.6277(27)
	Ningxia	0.6060(22)	0.6763(22)	0.7251(20)	0.6689(22)
	Xinjiang	0.6290(16)	0.6791(21)	0.7281(19)	0.6783(20)
Central	Shanxi	0.6384(14)	0.6995(13)	0.7401(16)	0.6946(13)
	Anhui	0.5966(25)	0.6599(26)	0.7061(25)	0.6555(25)
	Jiangxi	0.6048(23)	0.6699(23)	0.7099(24)	0.6669(23)
	Henan	0.6223(18)	0.6810(20)	0.7160(22)	0.6758(21)
	Hubei	0.6251(17)	0.6981(15)	0.7471(13)	0.6926(14)
	Hunan	0.6163(20)	0.6878(19)	0.7377(17)	0.6824(19)
	Mean	0.6322	0.6944	0.7374	0.6896
	Standard deviation	0.0630	0.0581	0.0571	0.0601

Referring to the construction of *HDI* and the actual situation in China, we selected 17 indicators to comprehensively measure environmental pollution for six dimensions: wastewater, waste gas, industrial solid waste, air quality, domestic waste treatment, and environmental self-purification rate (Table 2). Eqs (16) and (17) shows the specific calculation method.

**Table 2:** Indicators for the construction of *EDI*.

Dimensions	Indicators	Number
Wastewater	discharge of wastewater per capita^#^; discharge of COD per capita^#^; discharge of ammonia and nitrogen per capita^#^	3
Waste gas	emission of sulfur dioxide per capita ^#^; emission of smoke (dust) per capita^#^	2
Industrial solid waste^4^	per capita discharge of industrial solid waste^#^; per capita discharge of industrial solid hazardous waste^#^; the comprehensive utilization rate of industrial solid waste^^^	3
Air quality	the density of inhalable particles ^#^; the density of sulfur dioxide^#^; the density of carbon dioxide ^#^; days when air quality reaches or is better than level II^^^	4
Domestic waste treatment	harmless treatment rate of domestic waste^^^: per capita domestic waste removal volume ^^^	2
Environmental self-purification rate	annual forest coverage^^^; green area per capita^^^; water resources per capita^^^	3
Total		17

Note: Air quality is measured by the statistical indicators of major cities in each province (city) due to the limit of the availability of data; (#) and (^) represent the use of Eqs (14) and (15), respectively. The industrial waste water and industrial waste gas emissions of each province (city) are not recorded after 2015 as the result of changes in statistical caliber, so this paper uses waste water and waste gas emissions to replace. Becauses of the change of statistical caliber, emission of smoke (dust) per capita for 2004–2010 is measured by per capita emission of industrial waste gas in each province (city) and the indicator for 2011–2017 is measured by per capita emissions of each province (city).

$$EX_{ij}=(X_{ij}-X_{i}^{*})/(X_{i}^{**}-X_{i}^{*})$$ (14)

or:

$$EX_{ij}=(X_{i}^{**}-X_{ij})/(X_{i}^{**}-X_{i}^{*})$$ (15)

$$EDIX_{ij}=1/n\sum_{i=1}^{n}EX_{ij}$$ (16)

$$EDI_{j}=1/6\sum_{i=1}^{6}EDIX_{ij}$$ (17)

*EX*_*ij*_, *EDIX*_*ij*,_ and *EDI*_*j*_ represent the indicator value of dimension *i*, the value of dimension *i*, and the Environment Degradation Index (*EDI*) of province (city) *j*, respectively.

Guizhou, Yunnan, Qinghai, and Gansu had the lowest level of human development, and their rankings for environmental quality were 9, 5, 25, and 24, respectively (Table 3). Although the human development level for Yunnan and Guizhou was relatively low, their environmental quality was the best in China. This might have been because the industrialization process in the two provinces was relatively undeveloped, and so the damage to the environment was also small. Beijing, Shanghai, Tianjin, Jiangsu, and Liaoning ranked in the top five in terms of *HDI* with their *EDI* rankings of 1, 6, 13, 8, and 22, respectively. Those provinces (cities) were highly industrialized, but the rankings of their *EDI* were not in the top five except for Beijing. Industrialization may be one of the factors that led to the deterioration of the environment.

**Table 3:** The description of the Environment Degradation Index (*EDI*) and its sub-indicators (average value from 2004 to 2017) for 29 provinces in China.

Regions	Provinces	Wastewater	Waste gas	Industrial solid waste	Air quality	Domestic waste treatment	Self-purification rate of the Environment	EDI
North-east	Liaoning	0.5856(27)	0.4014(24)	0.4810(27)	0.4814(18)	0.4446(07)	0.6740(10)	0.5113(22)
	Jilin	0.4461(16)	0.2282(17)	0.2605(16)	0.3260(07)	0.6616(26)	0.7056(12)	0.4380(19)
	Heilongjiang	0.5006(23)	0.2531(20)	0.2114(14)	0.4606(16)	0.6772(28)	0.6313(06)	0.4557(21)
East	Beijing	0.2589(07)	0.0081(01)	0.1356(09)	0.5933(26)	0.0394(01)	0.6354(07)	0.2784(01)
	Tianjin	0.4481(17)	0.1673(12)	0.0516(01)	0.4957(19)	0.3993(05)	0.8817(26)	0.4073(13)
	Hebei	0.2640(08)	0.3276(22)	0.3811(25)	0.6197(27)	0.6484(25)	0.8748(25)	0.5193(23)
	Shanghai	0.6870(28)	0.1020(03)	0.0865(05)	0.3562(09)	0.2448(02)	0.7199(13)	0.3661(06)
	Jiangsu	0.4905(21)	0.1474(10)	0.0705(03)	0.4730(17)	0.3896(04)	0.7534(18)	0.3874(08)
	Zhejiang	0.4896(20)	0.1233(08)	0.0644(02)	0.4581(15)	0.3023(03)	0.5602(03)	0.3330(04)
	Fujian	0.5214(24)	0.1139(05)	0.1708(12)	0.1221(01)	0.4432(06)	0.5250(02)	0.3161(02)
	Shandong	0.2885(10)	0.1965(15)	0.1304(08)	0.5624(25)	0.4659(09)	0.8432(24)	0.4145(14)
	Guangdong	0.5308(25)	0.0603(02)	0.0749(04)	0.3391(08)	0.4528(08)	0.4545(01)	0.3187(03)
West	Inner Mongolia	0.4714(19)	0.8787(28)	0.5183(28)	0.3704(10)	0.5198(13)	0.7831(20)	0.5903(27)
	Guangxi	0.5401(26)	0.2218(16)	0.2314(15)	0.1741(02)	0.5910(16)	0.5799(04)	0.3897(10)
	Chongqing	0.3572(13)	0.2413(19)	0.1299(07)	0.4395(14)	0.4868(10)	0.7415(15)	0.3994(12)
	Sichuan	0.2737(09)	0.1119(04)	0.2993(17)	0.5129(20)	0.5794(15)	0.7323(14)	0.4182(15)
	Guizhou	0.0733(01)	0.3396(23)	0.3557(22)	0.1917(03)	0.6226(22)	0.7481(16)	0.3885(09)
	Yunnan	0.1070(02)	0.1288(09)	0.3549(21)	0.2091(04)	0.6348(24)	0.6561(09)	0.3484(05)
	Shaanxi	0.2214(04)	0.2984(21)	0.3076(19)	0.5442(22)	0.5668(14)	0.7670(19)	0.4509(20)
	Gansu	0.1859(03)	0.2389(18)	0.3432(20)	0.6527(28)	0.8639(29)	0.9419(29)	0.5378(24)
	Qinghai	0.4158(14)	0.5614(26)	0.8488(29)	0.3844(12)	0.4898(11)	0.6499(08)	0.5584(25)
	Ningxia	0.7399(29)	0.9012(29)	0.3020(18)	0.3713(11)	0.5094(12)	0.8049(23)	0.6048(29)
	Xinjiang	0.4536(18)	0.5435(25)	0.3656(24)	0.7278(29)	0.6188(20)	0.7853(21)	0.5824(26)
Central	Shanxi	0.2989(11)	0.7340(27)	0.4416(26)	0.5482(23)	0.6320(23)	0.9013(28)	0.5927(28)
	Anhui	0.2510(06)	0.1165(06)	0.1136(06)	0.3124(06)	0.6641(27)	0.7985(22)	0.3760(07)
	Jiangxi	0.3468(12)	0.1728(14)	0.3608(23)	0.2818(05)	0.6198(21)	0.5811(05)	0.3939(11)
	Henan	0.2323(05)	0.1662(11)	0.1608(10)	0.5489(24)	0.6088(18)	0.8950(27)	0.4353(18)
	Hubei	0.4425(15)	0.1176(07)	0.1690(11)	0.5354(21)	0.5971(17)	0.7490(17)	0.4351(17)
	Hunan	0.4912(22)	0.1677(13)	0.1721(13)	0.4053(13)	0.6111(19)	0.6936(11)	0.4235(16)

Note: Relative rankings are in parentheses (in reverse order of *EDI)*: The higher the *EDI*, the greater was the pollution, and the lower was environmental quality and ranking. Hainan and Tibet province are not included in the sample due to a lack of statistical data.

**Fig 1:**
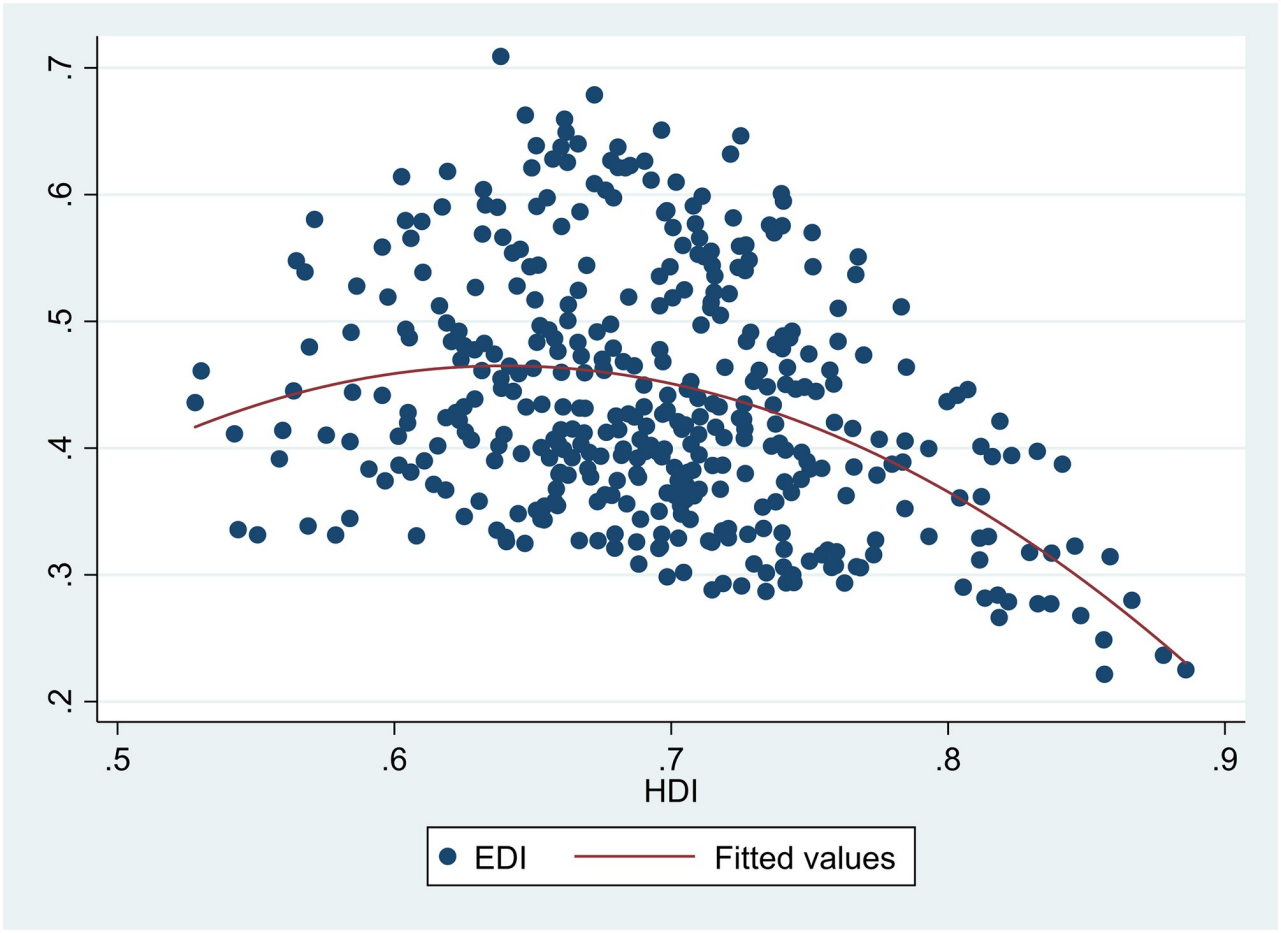
The relationship between the Human Development Index (*HDI*) and Environment Degradation Index (*EDI*) in China from 2004 to 2017.

**Fig 2:**
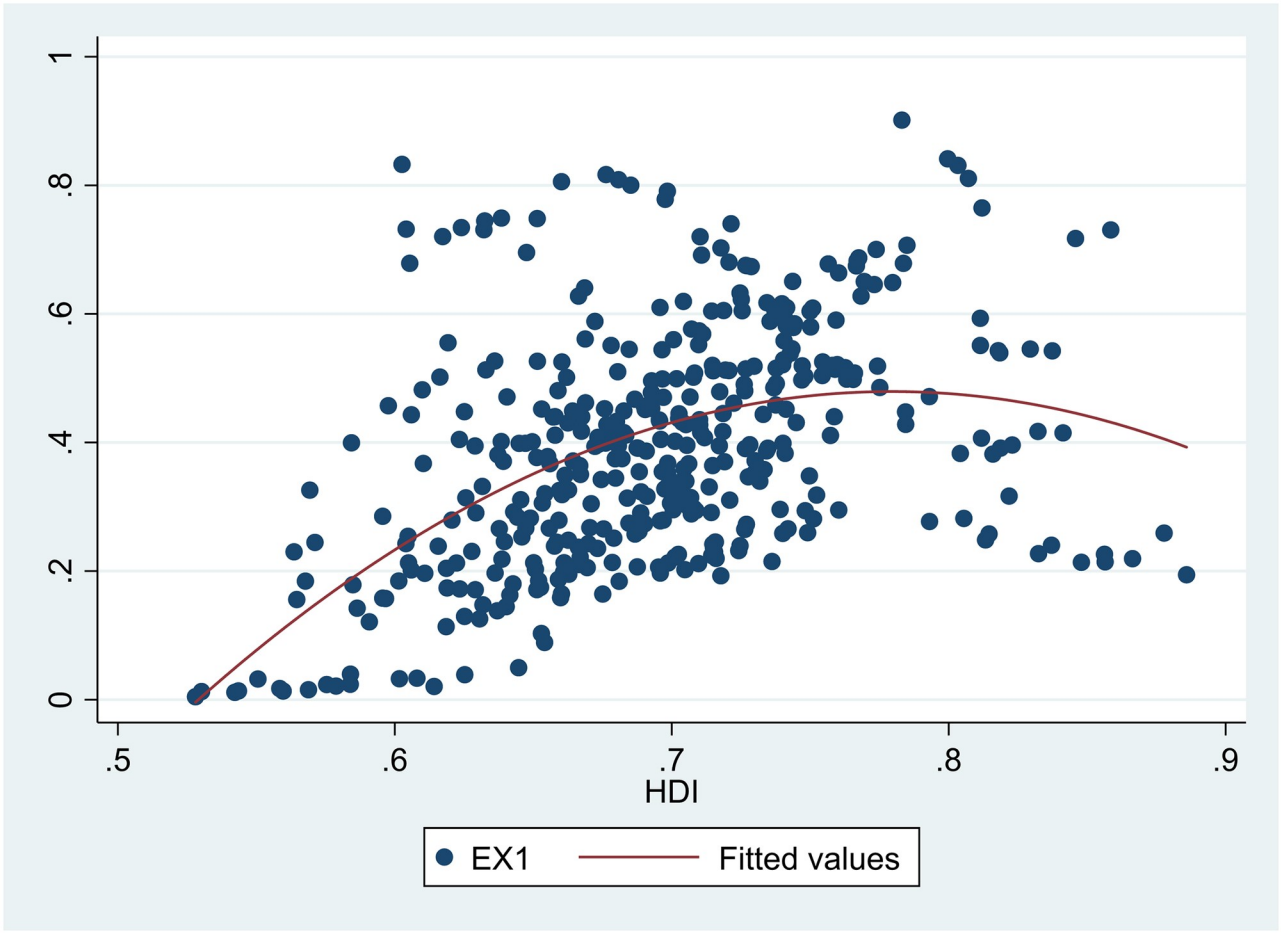
The relationship between the Human Development Index (*HDI*) and wastewater (*EX1*) in China from 2004 to 2017.

**Fig 3:**
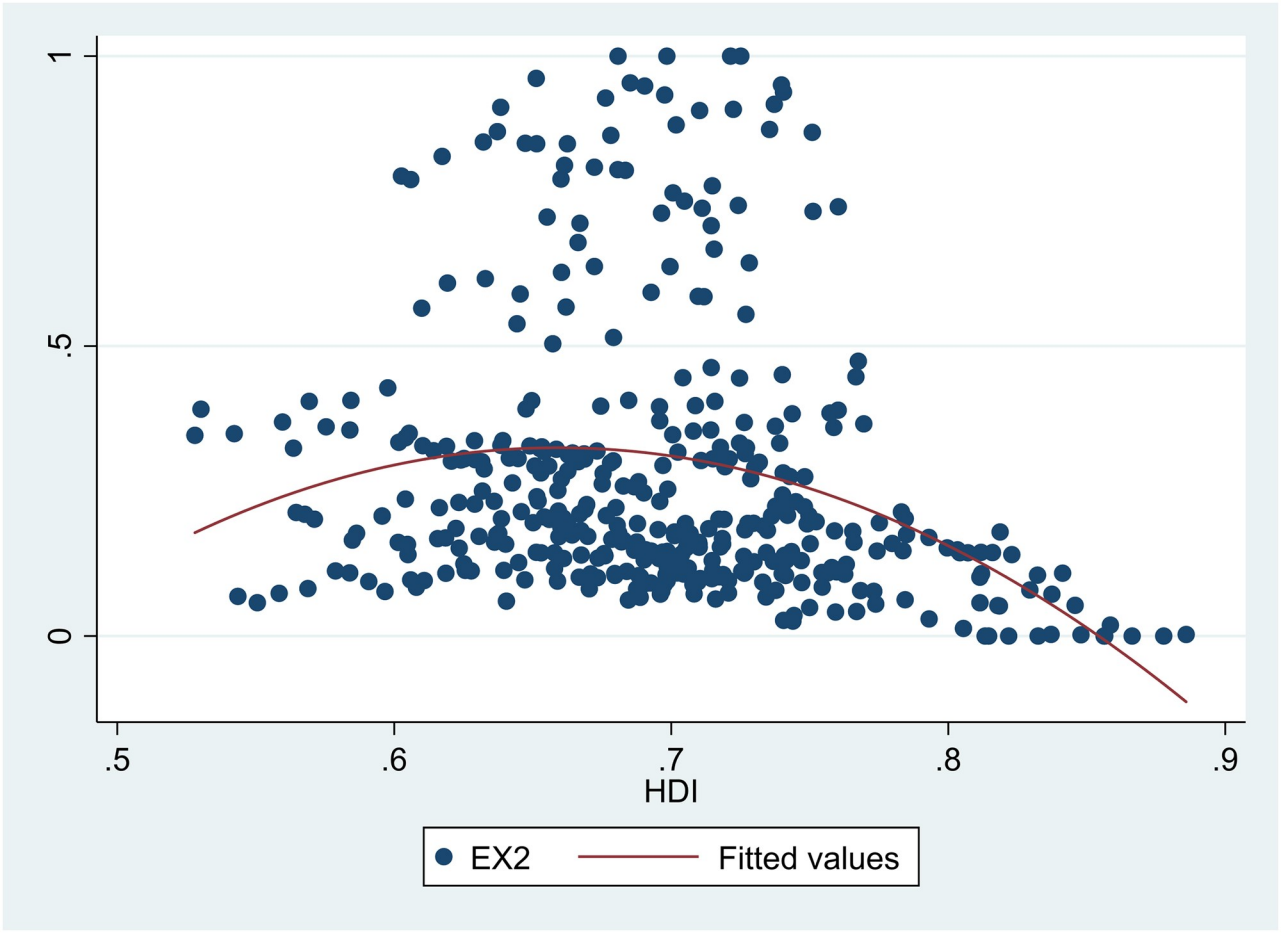
The relationship between the Human Development Index (*HDI*) and waste gas (*EX2*) in China from 2004 to 2017.

**Fig 4:**
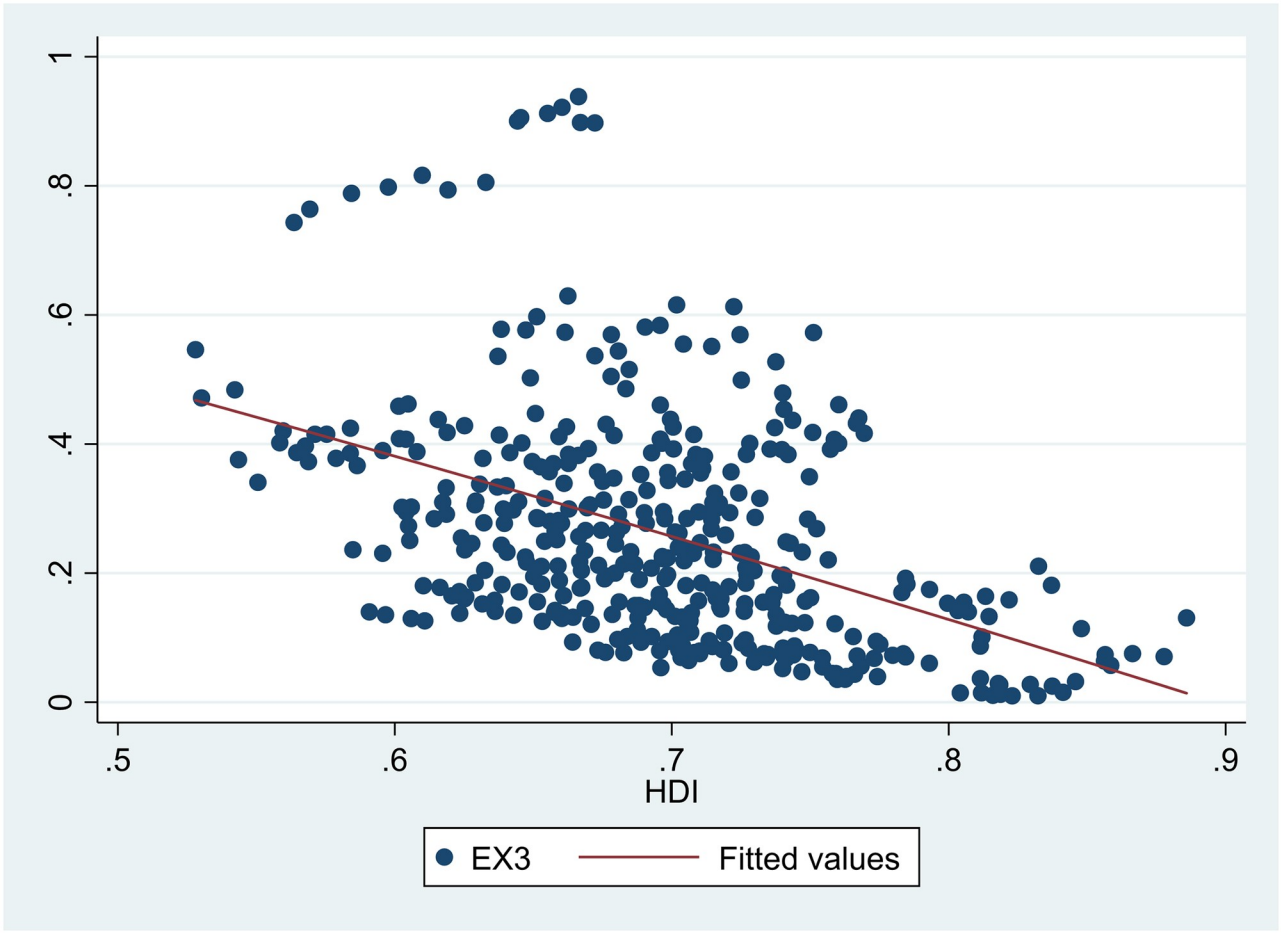
The relationship between the Human Development Index (*HDI*) and industrial solid waste (*EX3*) in China from 2004 to 2017.

**Fig 5:**
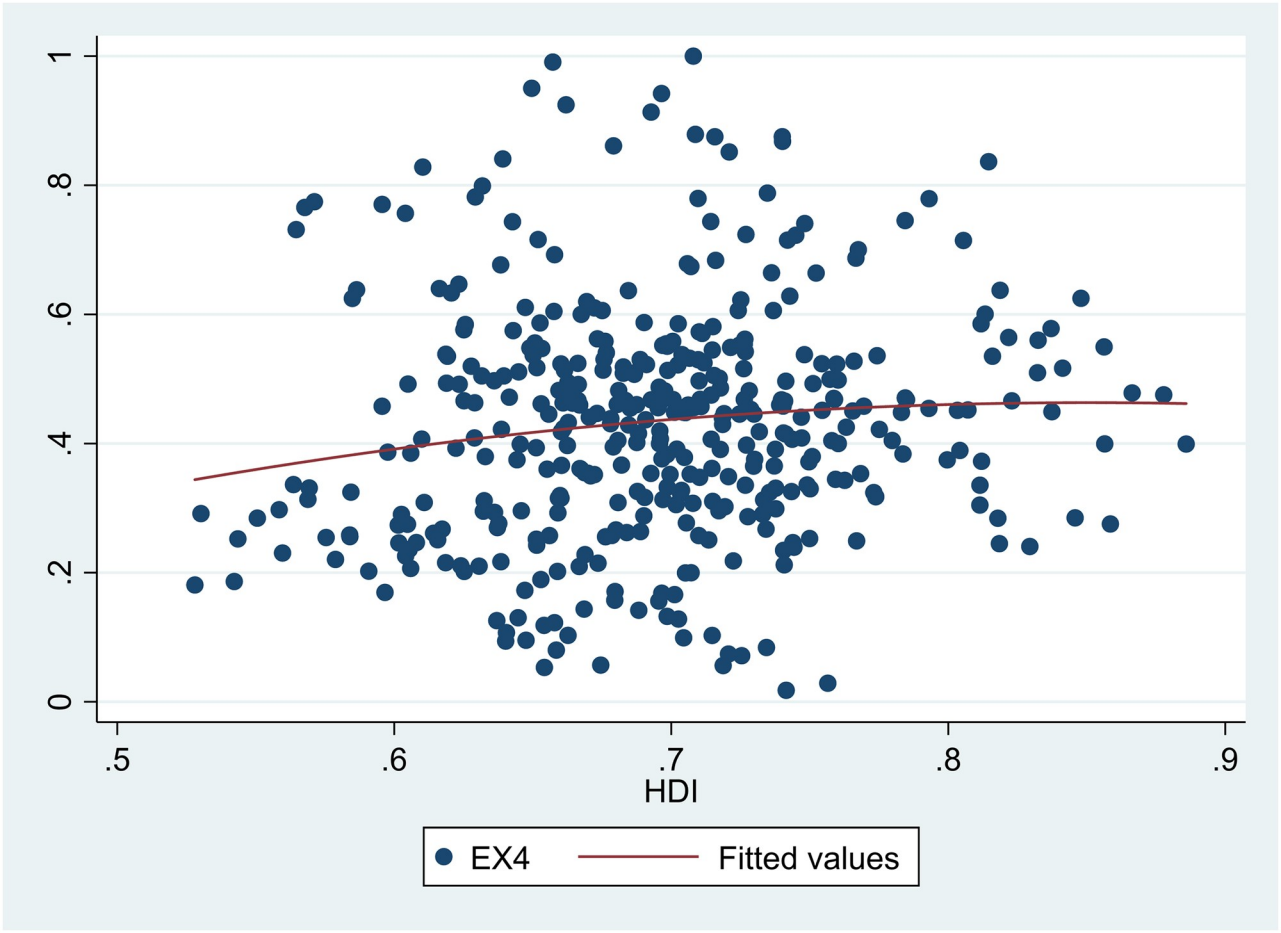
The relationship between the Human Development Index (*HDI*) and air quality (*EX4*) in China from 2004 to 2017.

**Fig 6:**
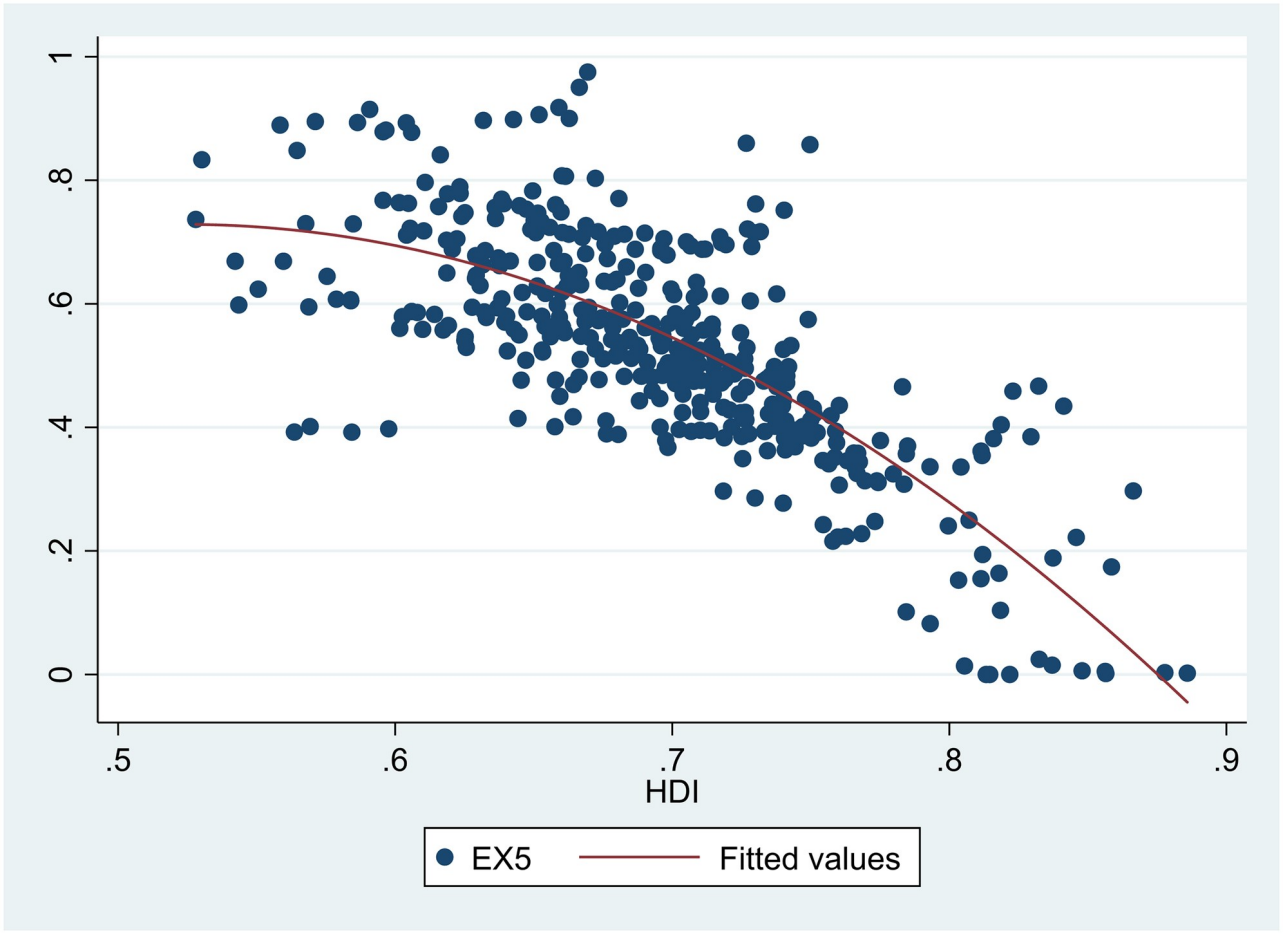
The relationship between the Human Development Index (*HDI*) and domestic waste treatment (*EX5*) in China from 2004 to 2017.

**Fig 7:**
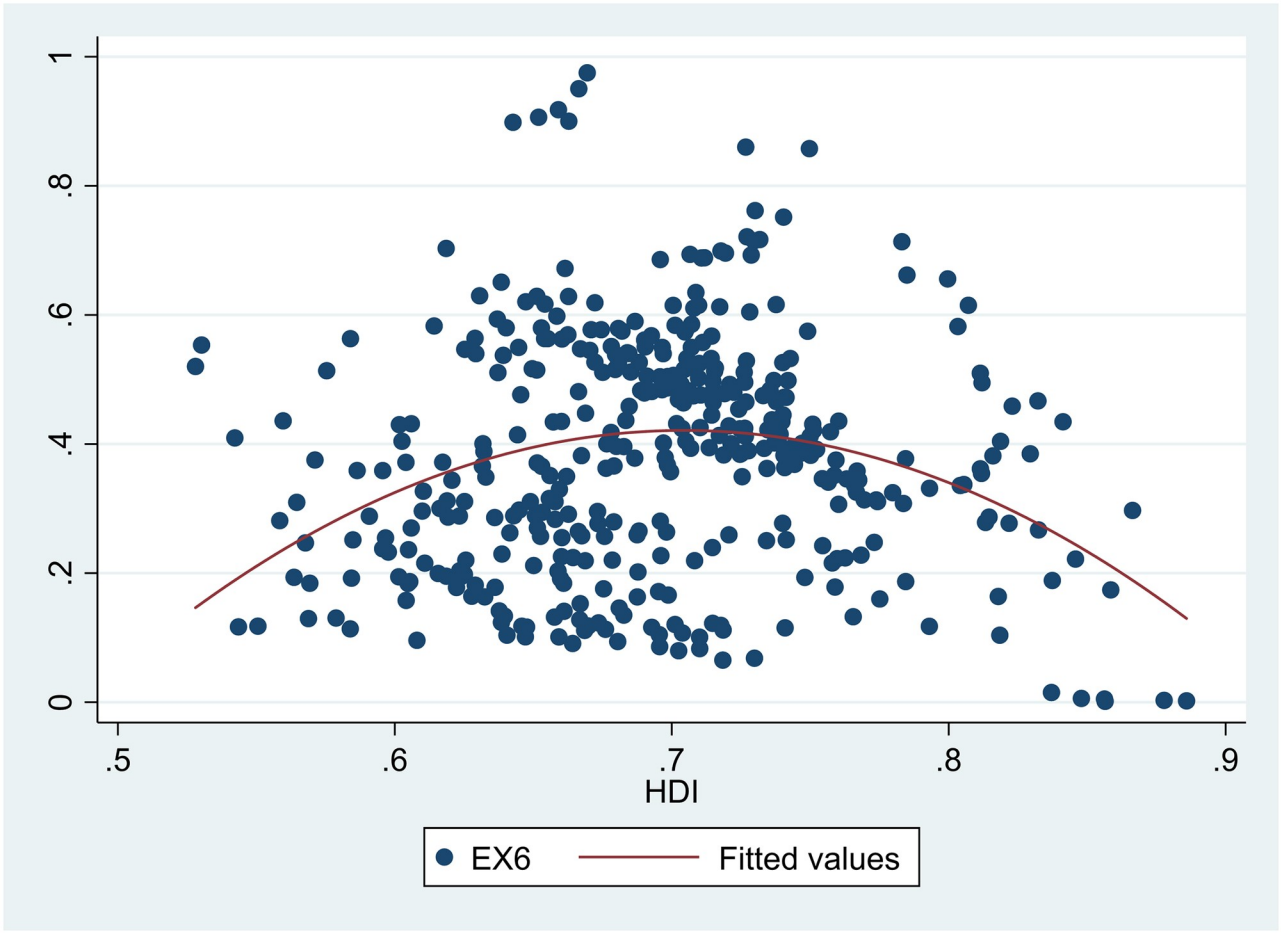
The relationship between the Human Development Index (*HDI*) and environmental self-purification rate (*EX6*) in China from 2004 to 2017.

A scatter diagram between *HDI* and *EDI* 7 showed there was a non-linear relationship between them (Fig 1). Besides, scatter diagrams showed there were nonlinear relationships between *HDI* and the six sub-dimensions of *EDI* (Figs 2–7).

This study did not include Hainan and Tibet provinces due to a lack of data for the two provinces. Thus, our empirical analysis was based on data for 29 Chinese provinces (cities) from 2004 to 2017. The data used in this study were from the *China Statistical Yearbook*, *China Environmental Yearbook*, *China Demographic Yearbook*, *China Foreign Economic Statistics Yearbook*, and the National Bureau of Statistics (http://www.stats.gov.cn).

## 4 Results and discussion

### 4.1 Estimation results

For Eq (1), the estimated values of the first and second powers of *HDI* were 5.2781 and -2.3476 with a significance level of 1% (Table 4), which proved that there was an inverted U-shaped relationship between China’s environmental pollution and human development. The results indicated that increasing investment in human development had a direct and significant effect on China’s environmental quality. Meanwhile, the estimation results of the six sub-indicators of *EDI*, which included *EDIX1*, *EDIX2*, *EDIX3*, *EDIX4*, *EDIX5*, *and EDIX6* that stood for wastewater, waste gas, industrial solid waste, air quality, domestic waste treatment, and self-purification rate of the environment, respectively, also showed significant correlations with *HDI* (Table 4). There was an inverted U-shaped relationship between *HDI* and exhaust gas, domestic waste treatment, and environmental self-purification rate. However, the relationship between *HDI* and wastewater was significantly negative, that is, the increase in *HDI* was favorable for reduction of wastewater discharge. The possible explanation is that in recent years, China has introduced the concept of cleaner production and has been strictly controlling sewage discharge rates. Also, local governments have been actively building sewage treatment systems. In December 2016, the Development and Reform Commission, and the Ministry of Housing and Urban-rural Development jointly issued the 13th Five-Year Plan for the Construction of National Urban Sewage Treatment and Recycling Facilities, which required that by the end of 2020, the urban sewage treatment rate should reach 95%, and this goal had been reached in 2017. Thus, with strict regulations, the relationship between wastewater and human development may have crossed the turning point of the inverted U-shaped curve.

**Table 4:** Estimation results of the simultaneous equations.

Eq (1)	EDI	Wastewater		Waste gas	Industrial solid waste		Air quality	Domestic waste treatment	Environmental self-purification rate
	EDI	EDIX1		EDIX2	EDIX3		EDIX4	EDIX5	EDIX6
HDI_it_	5.2781 (5.00)***	-2.6139 (-1.82)*		9.5035 (3.43)***	2.6085 (6.59)***		2.8004 (1.68)*	3.3595 (2.29)**	17.3024 (7.02)***
HDI_it_^2	-2.3476 (-3.33)***	0.2559 (0.26)		-4.4012 (-2.35)***			0.5448 (0.53)	-1.8047 (-2.01)**	-9.1850 (-5.62)***
Growth_it_	-0.1832 (-10.08)***	0.3287 (9.74)***		-0.2097 (-4.58)***	-0.3062 (-9.68)***		-0.2822 (-6.39)***	-0.2814 (-9.69)***	-0.3579 (-9.01)***
Struc_it_	0.0021 (3.48)***	0.0002 (0.24)		0.0061 (3.73)***	0.0015 (1.81)*		-0.0010 (-1.14)	0.0001 (-0.21)	-0.0028 (-2.13)**
Inst_it_	0.0001 (3.42)***	-0.0000 (-0.96)		0.0001 (1.41)	0.0001 (3.35)***		9.14e-06 (0.25)	0.00002 (0.96)	0.0001 (1.71)*
Fore_it_	-0.0100 (-4.09)***	-0.0061 (-1.82)*		-0.0267 (-4.10)***	-0.0119 (-12.96)***		-0.0100 (-2.82)***	-0.0045 (-1.79)*	-0.0284 (-5.31)***
Tech_it_	-0.0440 (-10.33)***	-0.0126 (-2.19)**		-0.1406 (-12.83)***	-0.0685 (-8.42)***		-0.0288 (-4.94)***	-0.0082 (-1.85)*	-0.0572 (-6.31)***
Constant	-0.1090 (-0.35)	-1.3639 (-2.78)***		-1.6926 (-2.05)**	1.8209 (15.18)***		1.3042 (2.93)***	2.0154 (5.44)***	-3.0632 (-4.26)***
Chi^2	320.01	282.92		316.44	108.82		211.30	837.30	148.66
R^2	0.38	0.12		0.43	0.11		0.56	0.48	0.13
Eq (2)	Growth								
EDI_it_	-3.1503 (-11.54)***								
EDIX1_it_		3.7819 (14.81)***							
EDIX2_it_			-0.6968 (-5.84)***						
EDIX3_it_						-1.9005 (-9.20)***			
EDIX4_it_							-4.9022*** (-17.31)		
EDIX5_it_								-3.4895 (-13.57)***	
EDIX6_it_									-1.2149 (-5.24)***
H_it_	0.2241 (8.05)***	0.2278 (5.72)***	0.2461 (8.68)***			0.2381 (7.19)***	0.4386 (12.25)***	0.0792 (2.04)**	0.3357 (11.11)***
k_it_	0.3787 (14.30)***	0.1481 (5.28)***	0.4138 (14.56)***			0.2846 (10.40)***	0.4064 (9.58)***	0.1032 (4.77)***	0.3418 (10.55)***
Constant	9.4937 (30.95)***	6.7197 (23.42)***	8.1007 (32.17)***			8.5430 (27.25)***	8.3419 (22.95)***	11.3805 (25.30)***	7.6286 (26.31)***
Chi^2	1384.97	736.51	1299.91			974.72	814.13	978.29	907.52
R^2	0.75	0.13	0.76			0.65	0.24	0.55	0.66
Obs	406	406	406			406	406	406	406

Note: All the models were significant at the 1% significance level (F test); Z statistical values are in brackets; ***, **, * indicate the significance level of 1%, 5%, and 10%, respectively.

Also, *HDI* was positively correlated with industrial solid waste and air quality; the rise in *HDI* intensified industrial solid waste pollution and air pollution. With the expansion of industrialization, the treatment of industrial solid waste became more difficult due to the complexity and diversity of wastes. Although China’s comprehensive utilization rate of industrial solid waste increased gradually, there is still much room for improvement. For example, the comprehensive utilization rate in 2017 was 54%, with an additional utilization space of 46%. Meanwhile, although people were more concerned about air pollution in recent years, the improvement in air quality still faced great challenges due to the complex and long-term task of controlling air pollution. Therefore, the relationship among industrial solid waste, air quality, and *HDI* occurred on the left half of the inverted U-shaped curve.

The estimated results for the control variables for Eq (1) showed that data fit well with the model and most of the estimated coefficients were significant at the 10% significance level. For *Growth*, the estimated coefficients for *EDI*, *EDIX1*, *EDIX2*, *EDIX3*, *EDIX4*, *EDIX5*, *and EDIX6* were -0.1832, 0.3287, -0.2097, -0.3062, -0.2822, -0.2814, and -0.3579, respectively, and all of them were significant at the 1% level. Thus, we concluded that China was on the right half of the EKC curve that was composed of GDP per capita and environmental pollution, which meant that environmental quality improved with the increase in GDP per capita.

Industrial structure (*Struct*) was positively correlated with *EDI*. That is, with the expansion of industrialization, environmental quality deteriorated. However, within the six sub-indicators, only waste gas, industrial solid waste, and environmental self-purification rates were significantly correlated to industrial structure. Among them, the estimated coefficients of *Struct* for waste gas and industrial solid waste were 0.0061 and 0.0015, respectively, with *P* = 10%. This result was consistent with our previous analysis that provinces with a large proportion of the secondary industry, such as Shanxi (0.5335), Henan (0.5332), Shaanxi (0.5280), Shandong (0.5260), and Qinghai (0.5254), also faced relatively higher environmental pressure, with *EDI* rankings of 28, 18, 20, 14, and 25, respectively. Our results confirmed most of the EKC studies [7] and indicated that industrialization was an important factor that influenced regional environmental pollution. However, there was no significant correlation found between industrial structure and air quality or domestic waste treatment. The reason may be that the impact of industrial structure on environmental pollution mainly suggested that it was industrial pollution, so impacts on other forms of pollution were not significant.

Foreign trade (*Fore*) significantly improved overall environmental quality and its six sub-indicators, and regression coefficients for *EDI*, *EDIX1*, *EDIX2*, *EDIX3*, *EDIX4*, *EDIX5*, *and EDIX6* were -0.0100, -0.0061, -0.0267, -0.0119, -0.0100, -0.0045, and -0.0284, respectively. The outcomes confirmed the analysis of Copeland and Taylor [46], which indicated that as a result of technology spillover effects from foreign investment, foreign trade may not necessarily lead to deterioration of environmental quality in developing countries.

The regression coefficients for technological progress (*Tech*) in the seven regression equations were -0.0440, -0.0126, -0.1406, -0.0685, -0.0288, -0.0082 and -0.0572, respectively, at the significance level of 10%. Technological progress, without exception, promoted the improvement of regional environmental quality in China, which indicated that increasing scientific research funding was an effective way to improve environmental quality. Meanwhile, we found that the effect of pollution control intensity (*Inst*) on the improvement in environmental quality was minimal, which indicated that improving environmental quality through investment in R&D beforehand was better than investment in pollution abatement afterward.

The estimated results for Eq (2) suggested that the investment of material capital (*k*) and the accumulation of human capital (*H*) significantly promoted regional economic growth in China. The coefficients for both *k* and *H* were significant at the level of 5%. In the seven equations (*EDI*, *EDIX1*, *EDIX2*, *EDIX3*, *EDIX4*, *EDIX5*, *and EDIX6*), the coefficients for *k* were 0.3787, 0.1481, 0.4138, 0.2846, 0.4064, 0.1032, and 0.3418 and the coefficients for *H* were 0.2241, 0.2278, 0.2461, 0.2381, 0.4386, and 0.0792, respectively. The rise in educational level and the increase in knowledge-based talents brought by human development were critical to regional economic growth. In addition, comprehensive environmental pollution (*EDI*), waste gas (*EDIX2*), industrial solid waste (*EDIX3*), air quality (*EDIX4*), domestic waste pollution (*EDIX5*), and environmental self-purification rate (*EDIX6*) all had a significant negative impact on economic growth, with their coefficients being -3.1503, -0.6968, -1.9005, -4.9022, -3.4895, and -1.2149, respectively. In contrast, the coefficient of wastewater (*EDIX1*) was 3.7918, which indicated a positive effect on economic growth. In general, the deterioration in environmental quality significantly delayed China’s regional economic growth, and the negative effects showed a certain degree of robustness. However, this study did not analyze the detailed mechanism of action for these impacts, which should be studied further.

### 4.2 Sensitivity analysis

In this section, we changed the calculation method for *HDI* to test the robustness of the results. Specifically, we measured the human development level with the *HDI* first proposed by UNDP in the *1990 Human Development Report*, which took the arithmetic average of the following three dimensions: i) life expectancy, which was measured by life expectancy at birth, ii) knowledge, which was measured by adult literacy rate (with a weight of 2/3) and the comprehensive enrollment rate for primary, intermediate, and advanced education (with a weight 1/3), and iii) resource acquisition, which was measured by GDP per capita. To avoid the multicollinearity problem that may occur in the econometric model, we eliminated the resource acquisition dimension following Costantini and Monni [25] and only utilized the longevity and knowledge dimension to measure human development level of the regions. According to the Human Development Report, life expectancy at birth ranges from 25 to 85 years; the adult literacy rate and the comprehensive enrollment rate of primary school, secondary school, and university range from 0% to 100%, with the adult literacy rate being the proportion of literate persons aged 15 and over in the total population aged 15 and over. In addition, because the data for the total number of people aged 6 to 22 is difficult to obtain during the sample period, we used the proportion of enrolled students (ordinary primary schools, middle schools, high schools, secondary vocational schools, and colleges) to represent the comprehensive enrollment rate.

The relationship between *HDI* and *EDI* still had an inverted U-shape after we changed the measurement method for *HDI* (Table 5). Also, the positive effects of production factors and the negative effect of *EDI* on economic growth were also consistent with the previous analysis. Thus, we concluded that the main results of this paper were robust.

**Table 5:** Estimation results of the robustness test.

Eq (7)	EDI	Eq (10)	Growth
HDIM_it_	32.2372 (6.28)***	EDI_it_	-1.3481 (-5.56)***
HDIM_it_^2	-22.9876 (-6.37)***	H_it_	0.2574 (10.25)***
Growth_it_	0.1064 (5.70)***	K_it_	0.4025 (16.09)***
Struc_it_	0.0028 (4.21)***		
Inst_it_	0.0001 (1.94)*		
Fore_it_	-0.0014 (-0.49)		
Tech_it_	-0.0455 (-8.28)***		
Constant	-11.8218 (-6.20)***		8.4033 (31.22)***
Chi^2	248.13		1584.51
R^2	0.20		0.80
Obs	406		406

Note: All the models were significant at the 1% level (F test); Z statistical values are in brackets; ***, **, * indicate the significance level of 1%, 5%, and 10%, respectively.

## 5 Conclusions

This study verified the existence of an inverted U-shaped curve between environmental pollution and human development, and we discussed the influence of pollution on regional economic growth. Meanwhile, there were also significant correlations between the six sub-dimensions of *EDI* and *HDI*. For waste gas, domestic waste treatment, environmental self-purification rate, the shape of the curve was still an inverted U, and for industrial solid waste and air quality, the relationship was positive. For wastewater, the correlation was negative, which indicated that wastewater may have been on the right side of the inverted U-shaped curve. Although the relationships were slightly different, it is indisputable that the improvement in China’s human development played an important role in promoting pollution control and in improving environmental quality in the long run.

As a whole, China’s economic growth helped to improve regional environmental quality, and environmental deterioration significantly delayed regional economic growth. At the same time, physical capital investment and human capital accumulation were essential to regional economic growth; this emphasized the importance of education and human development.

We believe that the key to improving environmental quality is to speed up human development and surpass the turning point of the inverted U-shaped curve as soon as possible, and increasing investment in human development is vital to solving the dilemma among economic growth, resource depletion, and environmental degradation in China.

## Supporting information

S1 File Data. (XLSX)
